# Field-Effect Transistor Based on Nanocrystalline Graphite for DNA Immobilization

**DOI:** 10.3390/biom15050619

**Published:** 2025-04-25

**Authors:** Bianca Adiaconita, Eugen Chiriac, Tiberiu Burinaru, Catalin Marculescu, Cristina Pachiu, Oana Brincoveanu, Octavian Simionescu, Marioara Avram

**Affiliations:** National Institute for Research and Development in Microtechnologies—IMT Bucharest, 126A Erou Iancu, Nicolae, 077190 Voluntari, Ilfov, Romania; bianca.adiaconita@imt.ro (B.A.); tiberiu.burinaru@imt.ro (T.B.); octavian.simionescu@imt.ro (O.S.)

**Keywords:** field-effect transistor, graphene-related material, nucleobase, Dirac point, mobility, DNA technologies

## Abstract

In recent years, field-effect transistors (FETs) based on graphene have attracted significant interest due to their unique electrical properties and their potential for biosensing and molecular detection applications. This study uses FETs with a nanocrystalline graphite (NCG) channel to detect DNA nucleobases. The exceptional electronic properties of NCG, and its high surface area, enable strong π–π stacking interactions with DNA nucleobases, promoting efficient adsorption and stabilization of the biomolecules. The direct attachment of nucleobases to the NCG channel leads to substantial changes in the device’s electrical characteristics, which can be measured in real time to assess DNA binding and sequence recognition. This method enables highly sensitive, label-free DNA detection, opening up new possibilities for rapid genetic analysis and diagnostics. Understanding the interactions between DNA nucleobases and graphene-based materials is crucial for advancing genetic research and biotechnology, paving the way for more accurate and efficient diagnostic tools.

## 1. Introduction

DNA is the foundation material of biological heredity because it carries the genetic information used in controlling all biological processes [1,2]. The detection of abnormal concentrations of nucleic acids can be used for evaluating the occurrence and progress of different pathological conditions. Therefore, nucleic acid biomarkers could be employed for the early detection of disease. They can also be employed for lab-on-a-chip (LOC), point-of-care detection (POC), precision medicine, and personalized therapy. There are different types of nucleic acids present in body fluids, such as cell-free DNA (cfDNA) and circulating tumor DNA (ctDNA). Of the two, ctDNA is the most widely searched tumor-related element in clinical applications to detect minimal residual disease, disease recurrence, and targetable genomic alterations, and to detect and monitor the emergence of resistance mechanisms [3,4]. Nucleic acids can also be used for rapid pathogen detection, which is an emerging issue in the clinical, environmental, and food industry sectors. Multidrug resistant bacteria like Enterococcus faecium, Staphylococcus aureus, Klebsiella pneumoniae, Acinetobacter baumannii, Pseudomonas aeruginosa, Enterobacter species (ESKAPE), and some foodborne pathogens such as Listeria monocytogenes, Salmonella spp., and Campylobacter spp. are responsible for 700,000 deaths per year globally and have a significant economic impact [5]. Additionally, the recent COVID-19 pandemic has demonstrated that there is an urgent need for fast, accurate, and cheap diagnosis methods [6]. It has been estimated that health issues caused by ESKAPE organisms generated 55 billion USD in excess direct and societal costs per year in the United States (US). In the European Union (EU) and European Economic Area (EEA) countries, healthcare costs sum up to 1.1–1.5 billion EUR yearly [7]. The global economic impact of COVID-19 was estimated to be between 77 billion and 2.7 trillion USD in 2019 [8].

In the last few years, field-effect transistors (FETs) have attracted much attention in the area of bio-detection due to their sensitivity in detecting molecular interactions. The FET-based biosensor relies on a biomolecular recognition event at the FET gate [9,10,11,12]. During specific bio-recognition interactions, the electric charge distribution changes the surface density of the electric charge carriers and, therefore, changes the conductivity of the source–drain channel [10,11,12]. Graphene is considered an ideal material for the construction of FET biosensors because the bandgap can be tuned by modifying the surface. In a typical graphene-based FET architecture, graphene is deposited/transferred on a Si substrate with a 300 nm SiO_2_ layer. The doped Si substrate acts as a back gate, which induces a charge density at the surface and hence changes the Fermi energy level in the graphene layer [13]. So far, several reports have demonstrated that graphene-based FETs with electrolytic top gates can be effectively used for charged molecule detection [14,15,16,17]. Compared with electrochemical signal readout, graphene-based FETs utilize electrical sensing to exploit the change in resistivity, mobility, and electric charge distribution when nucleic acids adsorb on the surface of the source–drain channel of the FET, and their small or even nanoscale dimensions allow them to provide better sensing abilities. Thus, these properties make graphene-based FETs a promising instrument for interface studies. The development of the graphene–nucleic acid nano–bio interface is straightforward and easy to tailor due to the self-assembly properties of DNA molecules and the detection specificity that single-stranded DNA (ssDNA) or single-stranded RNA (ssRNA) molecules offer [9,18,19,20,21,22,23,24,25].

Theoretical studies indicate that DNA molecules attach to the graphene surface through π−π stacking interactions [26,27]. The calculated binding energies are almost identical for adenine, thymine, and cytosine. The hierarchy of binding energies is as follows: G > A ≈ T ≈ C > U (G—guanine, A—adenine, T—thymine, C—cytosine, U—uracil). The authors found that the calculated binding energies were correlated with the polarizabilities of the nucleobases, making polarizability the dominant source of interaction between nucleobases and graphene. Single-stranded DNA possesses a much higher binding capacity to graphene than double-stranded DNA. Shorter strands of DNA adsorb faster and bind more tightly to graphene’s surface [26,27]. However, these theoretical studies assumed perfectly flat graphene layers, which differ from the real behavior of graphene layers that naturally tend to roll to achieve structural stability. The appearance of curvatures in the graphene layer can significantly alter the electronic properties and the binding capacity of nucleobases. Theoretical calculations that investigate the binding capacity of nucleobases to curved graphene, such as carbon nanotubes (CNTs), have been performed using high-precision first principle calculations [28]. The results show that the binding energy increases as the curvature decreases and reaches a maximum for planar graphene. However, these studies are based on CNT structures, not on the nonplanarity of graphene. Another study has reported that nucleobases have a stronger binding affinity to graphene with larger radii of curvature compared with the smaller CNTs, as large-size graphene exhibits significant curvature, as observed by quantum chemical calculations and atomic force microscopy (AFM) measurements [29]. It has been reported that the interaction energy increases systematically as the size of the system expands [30]. The curvature may provide additional stability for nucleobase binding on the graphene surface. Studies also show that ssDNA binds to graphene mainly through π–π attachment interactions [31,32,33,34]. Zhao et al. performed molecular dynamics (MD) simulations to investigate the self-assembly of double-stranded DNA segments on graphene’s surface [35]. They observed that DNA can form two distinct types of assembly. Both of them use π–π stacking as the main binding force onto the graphene layer.

As nucleic acids have a charged phosphate backbone, graphene-based FETs are an ideal tool in nucleic acid/graphene interface property sensing, interfacial studies, and interface-based sensing applications [14,15,16,17,36]. DNA probes, aptamers, or CRISPR/Cas9 molecules have been used as bio-receptors for LOC devices [37,38,39]. Additionally, as DNA molecules can bind directly to graphene, DNA origami molecules can be directly attached to it to develop new types of biosensors or drug delivery platforms [40,41,42]. Wasfi et al. developed an FET device based on graphite oxide decorated with a trimetallic nanocluster of gold, silver, and platinum for real-time nucleic acid detection with a 1.28 nM limit of detection (LOD) [43]. Ganguli et al. demonstrated that crumpled gFETs can be used to detect E. coli DNA down to zeptomolar (zM) concentrations [44]. Chen et al. fabricated a DNA-functionalized graphene field-effect transistor for quantitation of vascular endothelial growth factor, with a 3.24 pg/mL LOD [24]. Hwang et al. used deformed monolayer graphene channels for the detection of nucleic acids with a detection sensitivity down to 600 zM in buffer and 20 aM in human serum sample [45].

The transfer characteristic curves of BioFET devices, particularly those utilizing graphene and graphene-related field-effect transistors (GFETs), are crucial for understanding their biosensing applications. Studies indicate that DNA immobilization significantly affects the electrical characteristics of these devices and highlight the importance of reducing defects and preserving graphene’s properties to maintain stable transfer characteristics during DNA hybridization detection [46]. Similarly, Jia and Ju used simulations to demonstrate how ssDNA immobilization and hybridization affect GFETs’ performance [47]. They found that DNA’s negative charge alters the Dirac point and modulates conductivity, supporting the device’s role in biosensing. The charge accumulation method enhances signal detection by directly transferring charges, improving signal-to-noise ratios [48]. Additionally, Selvarajan et al. modeled GFET transfer characteristics, confirming that doping and environmental factors influence electrical responses, which is vital for optimizing DNA detection [49].

As there are no published works that describe this, we propose to study the interaction of ssDNA probes with nanocrystalline graphite (NCG). In the current paper, we propose to use NCG for the sensing area of an FET device. NCG is an innovative 3D carbonic nanomaterial with excellent electric properties. Additionally, the FETs used in the current paper are a two-gate FET with a back gate and a front gate. By understanding and characterizing the attachment of the DNA samples and how it influences the behavior of the FETs, we hope to further advance the fabrication of NCG-based LOC- and POC-type devices for disease diagnosis and pathogen detection.

This study represents a groundbreaking advancement in the field, as it is the first to report the direct interaction between guanine, adenine, cytosine, and thymine, with NCG and its significant impact on the electrical conductivity of NCG. The NCG–guanine complex demonstrates the highest conductivity among the nucleobases tested, marking a significant leap in understanding the role of nucleobases in modifying graphene-based materials’ electronic properties. This pioneering research opens new avenues for the development of guanine-based biosensors, providing insights that could shape the future of bioelectronics and biosensing technologies.

## 2. Materials and Methods

The cleaning of FET devices, deposition of single-stranded DNA probes, incubation, and washing were carried out at room temperature in a laminar flow hood within an ISO6 cleanroom (class 1000) to minimize surface contamination.

The source–drain channel surface characteristics were evaluated after the immobilization of nucleobases through energy dispersive X-ray spectroscopy (EDX/EDS), at an acceleration voltage of 15 kV, using the Nova NanoSEM 630 system (FEI Company, Hillsboro, OR, USA), which is equipped with an EDX detector (EDAX TEAM™, AMETEK, Inc., Berwyn Pennsylvania, USA).

Structural characterization was performed using Raman spectroscopy, which provides information regarding the shape and type of chemical bonds. Raman spectra were acquired using a Witec Raman spectrometer (Alpha-SNOM 300 S, WiTec GmbH, Germany), utilizing a 532 nm diode-pumped solid-state laser with a maximum power of 145 mW. The incident laser spot size was approximately 1.0 μm, with two objectives attached (50× and 100×) to a Thorlabs MY100X-806 microscope.

Electrical characterization was carried out using the semiconductor characterization system (DC) with the Wafer Probing Station-4200-SCS/C/Keithley Easyprobe EP6/Suss MicroTec.

Single-stranded DNA samples were purchased from Integrated DNA Technologies. We used ssDNA with a length of 20 bases of the same type with 100 µM concentration. We named them poly G (GGG GGG GGG GGG GGG GGG GG), poly A (AAA AAA AAA AAA AAA AAA AA), poly C (CCC CCC CCC CCC CCC CCC CC), and poly T (TTT TTT TTT TTT TTT TTT TT).

### Fabrication of the Nanocrystalline Graphite-Based Field-Effect Transistor for Direct Immobilization of Single-Stranded DNA Probes

The NCG FET was fabricated using conventional photolithography on a 4-inch p-doped Si wafer. The wafers were cleaned in Piranha solution by immersion. The FET dimension was 4.7 mm^2^, with a sensing area of 100 μm × 100 μm.

The fabrication process began with p-doped silicon wafers, which serve as the substrate, cleaned by immersion in Piranha solution for 30 min. The Si wafers were subjected to a thermal oxidation process in an oxidation furnace at a temperature of 900 °C for 340 min. The NCG channel between the source and drain was synthesized via plasma-enhanced chemical vapor deposition (PECVD) at a substrate temperature of ≈ 900 °C, with a growth time of 75 min, in a gas flow of CH_4_ (60 sccm) and H_2_ (75 sccm). The NCG film is deposited on the full wafer surface and patterned via photolithography. A thick positive photoresist (AZ4562) is used to transfer the pattern from the photomask, as the NCG film is etched via reactive ion etching (RIE) in an O_2_ plasma. The removal of the photoresist after RIE takes place in acetone and isopropyl alcohol. The source and drain electrodes are fabricated/patterned via the lift-off process: a negative photoresist (LOR 5A) and a positive photoresist (AZ1518) are spin-coated onto the substrate, followed by UV exposure through a specific photomask. A thin film of Cr/Au (10/300 nm) is then deposited, and the lift-off process is carried out in acetone with ultrasonic treatment to remove both the metal and the photoresist, resulting in the formation of the source–drain electrodes. The front gate fabrication is also accomplished via a lift-off procedure: after the deposition of two sacrificial photoresists, followed by alignment and exposure to a photomask with the front gate design, a thin aluminum oxide (Al_2_O_3_) film, with a thickness of 10 nm, is deposited at 120 °C via atomic layer deposition (ALD). This film serves to isolate the gate contact. This is followed by the deposition of a 10 nm thick Cr layer and a 300 nm thick Au layer, with subsequent electrode patterning using the lift-off technique. The final step is dicing the wafers into individual sensors, according to the device geometry.

**Figure 1:**
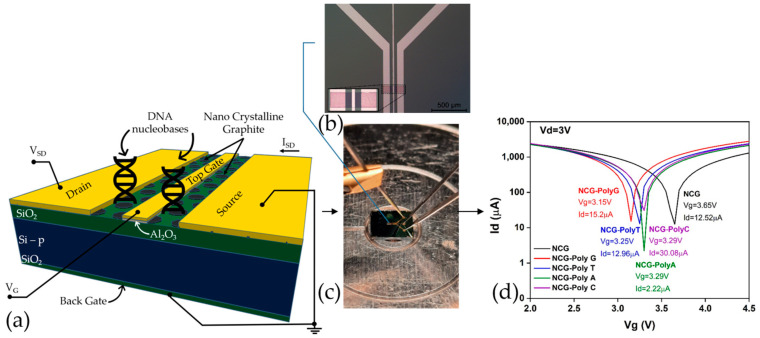
(**a**) Schematic representation of the NCG-based FET device depicting the NCG channel and the 4 electrodes. (**b**) Optical microscopy image of the NCG-FET at 5× magnification with an enhanced view of the NCG channel (inset). (**c**) Experimental setup for the electrical characterizations. (**d**) *I_d_*/*V_g_* plot for different nucleotide at *V_d_* = 3 V.

Figure 1a,b display a schematic representation of the functionalized NCG-based FET and an optical microscopy image of the FET device, respectively, while Figure 1c showcases the experimental setup for the electrical characterizations. The microfabrication process is more extensively presented in a previous article [50]. Figure 1d represents the transfer characteristic curves of an NCG-FET after the direct immobilization of the different DNA nucleobases (Poly G, poly T, poly A, and poly C). The immobilization of different nucleobases shifts the transfer curves, affecting the drain current (*I_d_*) and shifting the Dirac point—Poly G (Red) and poly T (Blue) show relatively similar shifts. These shifts occur due to the varying charge distribution and dipole moment of the DNA nucleobases interacting with the graphene surface, affecting the charge carrier concentration and transport properties of the NCG-FET. The shift direction and magnitude depend on the specific nucleobase’s electronic properties and its interaction with the graphene-based sensing layer.

## 3. Results

Understanding the nucleobase–graphene interactions is essential for advancements in genetic research and biotechnology, as they play a critical role in maintaining genetic accuracy and participating in various biological processes such as energy metabolism and cell signaling. Direct detection of these nucleobases on NCG surfaces represents a significant step toward the development of advanced biosensors. Due to its superior electrical properties, NCG provides an optimal platform for such applications. While existing literature primarily reports theoretical data on the direct interaction between graphene and nucleotide bases, our study presents novel experimental findings, demonstrating promising results for the direct detection of DNA. When integrated into an FET, this approach enables rapid and highly sensitive nucleobase detection, paving the way for high-performance biosensors with applications in genetics, medical diagnostics, and biotechnology.

### Direct Adsorption of Nucleobase on NCG

Based on the theory that DNA interacts with the graphene surface through various mechanisms, such as π–π attachment, hydrogen bonding, electrostatic interactions, van der Waals forces, and hydrophobic interactions, forming spherical particles in the case of poly A and poly C, while poly T and poly G form a network on the surface, the main mechanism we applied in the preliminary studies is free adsorption.

For the attachment of single-stranded DNA probes on the GFET, we aimed to functionalize the NCG channel non-covalently by utilizing π–π interactions between the graphene layers and the nucleotides. We chose this method to avoid altering the structure and properties of the graphene-based material. The hydrophobic interaction between the DNA bases and NCG demonstrated excellent nucleotide base adsorption capabilities without compromising their structural integrity.

First, each sample is cleaned with 3 µL of isopropyl alcohol. Then, the samples are dried at room temperature in a laminar flow hood for 10 min, and the ssDNA specimens are incubated. The immobilization process involves the π–π interaction between the NCG surfaces and the four nucleotide bases (G, A, T, C), each containing 20 nucleotides. We added 5 μL of a 100 µM solution—poly G/poly A/poly T/poly C—onto the surface of the NCG channel. A droplet formed above the channel, and the FET devices were kept at room temperature overnight in the hood. After incubation, the samples were cleaned three times with the specific nucleotide buffer IDTE: 1× TE solution, pH = 8, certified nuclease-free, and stored at room temperature to dry.

**Figure 2:**
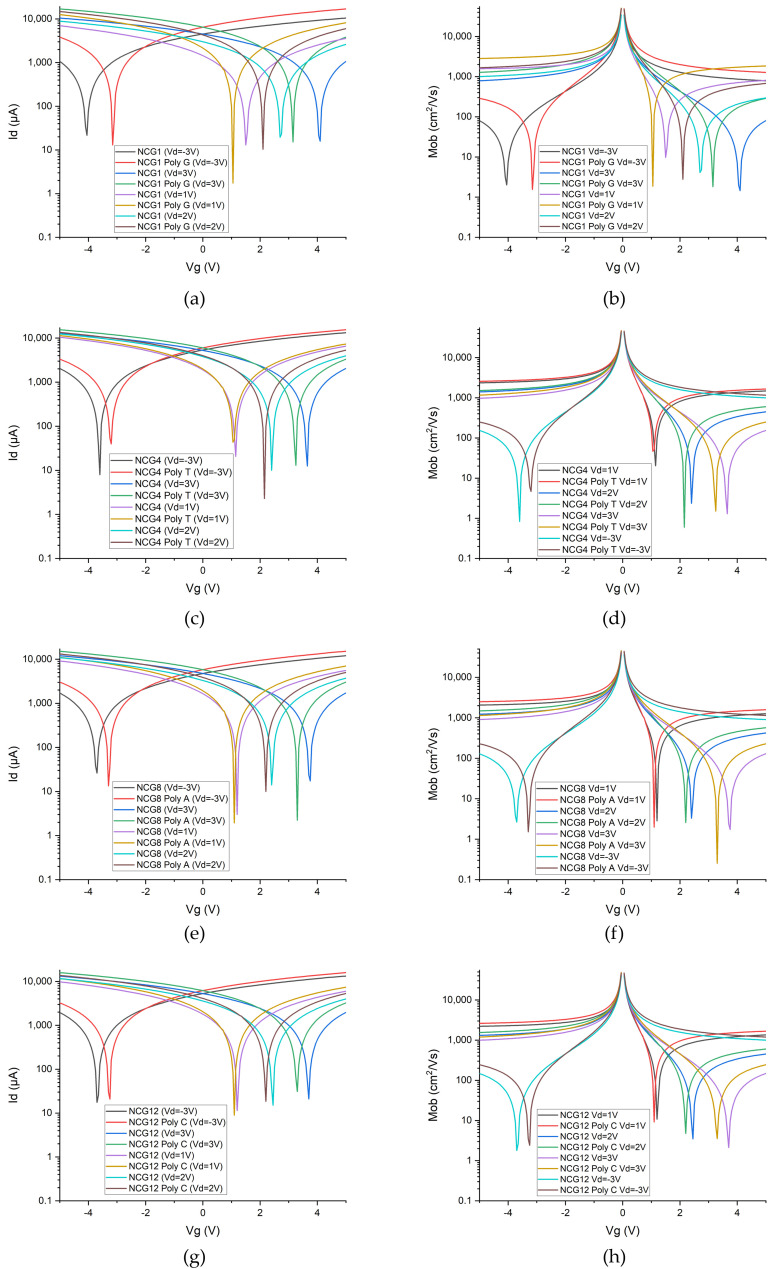
I–V characteristics of the NCG-FETs before and after (**a**) guanine, (**c**) thymine, (**e**) adenine, and (**g**) cytosine adsorption/direct interaction. Mobility of the NCG-FETs before and after (**b**) guanine, (**d**) thymine, (**f**) adenine, and (**h**) cytosine adsorption at a drain potential of (top-left) 1 V, (top-right) 2 V, (bottom-left) 3 V, and (bottom-right) −3 V.

The electrical response of the NCG-based FETs for the four types of nucleotides is presented in Figure 2. The transfer characteristics, where the gate voltage (*V_g_*) is swept in the interval [−5 V; 5 V]; the drain voltage is biased at −3 V, 1 V, 2 V, and 3 V; and the drain current is measured, are displayed in Figure 2a,c,e,g. Based on the presented transfer characteristics, the mobility is computed in each case and is showcased in Figure 2b,d,f,h.

The detection of DNA nucleobases using NCG-FETs is achieved by measuring the electrical response following the adsorption of ssDNA samples onto the NCG source–drain channels. The adsorption of nucleobases onto the FET channels induces a leftward shift of the Dirac point. This leftward shift is attributed to n-type doping of NCG, caused by π–π stacking interactions between NCG and the electron-rich nucleobases in DNA molecules. The corresponding values of the Dirac point and its respective shift after ssDNA adsorption are presented for each of the four nucleobases in Table 1. Depending on the applied gate voltage, the Dirac point shifts to the left by 0.45 V and 0.9 V in the case of guanine, by 0.1 V to 0.4 V in the case of thymine, by 0.1 V to 0.46 V in the case of adenine, and by 0.1 V and 0.45 V in the case of cytosine. The Dirac point shift—an indicator of sensitivity—is significant upon nucleobases adsorption compared with the reference NCG-FET.

From an EDAX perspective (Table 2), the adsorption of guanine, thymine, adenine, and cytosine onto the NCG channel is confirmed by the presence of carbon (C), oxygen (O), and nitrogen (N). Guanine exhibits significant charge transfer at the NCG interface, associated with high interaction energy due to π-π interactions playing a crucial role, as well as the presence of both amino (−NH_2_) and carbonyl (=O_6_) groups, which can interact with the π-electron cloud at the NCG surface. For thymine, the nitrogen signal intensity is lower than that observed for guanine, which is expected given that guanine is a stronger nitrogenous base. Conversely, an increase in oxygen presence is observed, as thymine contains two oxygen atoms in its chemical structure, whereas guanine has only one. Adenine shows a more balanced composition, with a notable increase in nitrogen, while in the case of cytosine, the nitrogen presence is lower, which is likely due to both the lower number of nitrogen atoms in cytosine (three nitrogen atoms) and the fact that cytosine is a weaker base.

**Figure 3:**
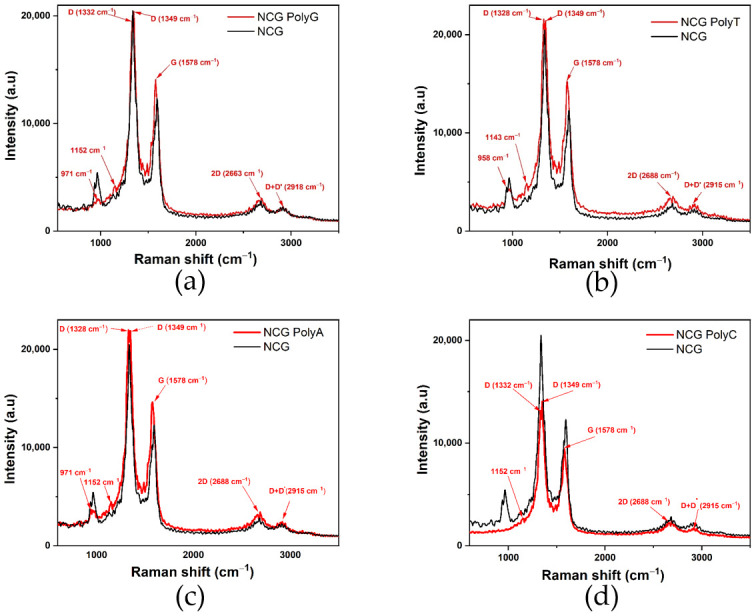
Comparative Raman spectra of the NCG-FETs before and after (**a**) poly G, (**b**) poly T, (**c**) poly A, and (**d**) poly C adsorption.

The presence of nucleobases can either enhance or attenuate NCG-specific Raman peaks (Figure 3), which is expected due to electrostatic interactions and hydrogen bond formation. The characteristic NCG Raman peaks, such as D (≈1350 cm⁻^1^), G (≈1580 cm⁻^1^), 2D (≈2680 cm⁻^1^), and D + D’ (≈2980 cm⁻^1^), may experience frequency shifts in the presence of nucleobases. These shifts indicate changes in the electronic structure or graphene lattice dynamics.

The Raman spectra acquired from the NCG-FETs after poly G, poly A, and poly C adsorption reveal the appearance of a 1152 cm⁻^1^ peak, while after poly T the new peak appears at 1143 cm⁻^1^. This peak is frequently associated with specific vibrational modes of chemical bonds in organic molecules, including nucleobases. This peak may be related to C–N bond vibrations common in nucleobase structures, C–C bond vibrations, especially within the aromatic ring, or C–H stretching modes. Additionally, D-band doubling is observed, which may indicate changes in the electronic structure. When the ssDNA is adsorbed onto the NCG surface, it influences the lattice vibrations, leading to D-band splitting. Initially, the D-band appeared at 1336 cm⁻^1^ for the control sample for poly G, poly T, and poly A, and at 1332 cm⁻^1^ for the control sample for poly C. After ssDNA adsorption, the D band splits into two peaks at 1332 cm⁻^1^ and 1349 cm⁻^1^ for poly G and poly C, 1328 cm⁻^1^ and 1349 cm⁻^1^ for poly T, and 1328 cm⁻^1^ and 1349 cm⁻^1^ for poly A. The G-band, initially detected at 1594 cm⁻^1^ in the pristine NCG channels, shifts to 1578 cm⁻^1^ after ssDNA attachment for all nucleobases. Peaks observed at 1489 cm⁻^1^ and 1529 cm⁻^1^ are associated with N–H bond vibrations and other vibrational modes of amino groups. Furthermore, N–H stretching vibrations appear at approximately 3125 cm⁻^1^ and 3248 cm⁻^1^.

The results from our experimental study support the conclusions drawn in previous theoretical studies from the literature, which suggest that the presence of guanine significantly enhances the electrical conductivity of pure graphene. Theoretical models indicate that this enhancement is due to guanine’s ability to donate electrons to graphene, thus facilitating n doping and promoting easier electron flow. Furthermore, the NCG–guanine complex exhibits the highest conductivity among the nucleobases tested, confirming its superior electrical properties as predicted by theoretical analyses.

## 4. Discussion

**Figure 4:**
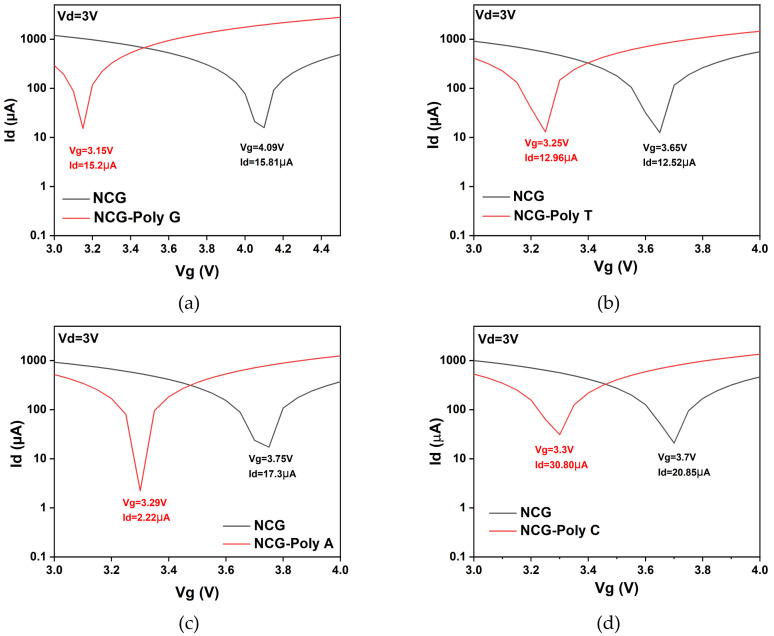
Transfer characteristic curves of the NCG-FET before and after (**a**) guanine, (**b**) thymine, (**c**) adenine, and (**d**) cytosine adsorption. Drain current (*I_d_*) vs. gate potential (*V_g_*) plot for the different nucleotides at a fixed drain potential of *V_d_* = 3 V.

The comparison of the four nucleobases (adenine, thymine, guanine, and cytosine) adsorbed onto the NCG-FETs reveals distinct differences in their interaction with the NCG surface. Figure 4 represents the transfer characteristic curves of the NCG-FET before and after the direct immobilization of different DNA nucleobases (poly G, poly T, poly A, and poly C) at a 3 V drain potential, which displayed the highest shift in the Dirac point. Poly G and poly T show relatively similar drain currents at the Dirac point. These shifts occur due to the varying charge distribution and dipole moment of the DNA nucleobases interacting with the NCG surface, affecting the charge carrier concentration and transport properties of the NCG-FET. The shift direction and magnitude depend on the specific nucleobase’s electronic properties and its interaction with the NCG-based sensing layer.

Both adenine and guanine, which are purine bases, exhibit the strongest interactions with NCG, resulting in significant n-doping effects. The Dirac point shift, a key indicator of doping, is most pronounced for these two bases, with shifts reaching up to 0.46 V for adenine and even greater for guanine. This is attributed to the electron-rich nature of purines, particularly their nitrogen atoms, which participate in π–π stacking interactions with the NCG surface. These strong interactions lead to significant changes in the electronic structure of NCG, as evidenced by the pronounced Dirac point shifts and enhanced electron flow through the NCG upon adsorption.

In contrast, thymine and cytosine, the pyrimidine bases, induce weaker electrical interactions with the NCG surface. The Dirac point shift in these bases is more moderate, ranging from 0.1 V to 0.4 V for thymine and up to 0.45 V for cytosine. These shifts indicate a weaker n-doping effect compared with purines, as these bases have fewer nitrogen atoms and exhibit less electron-donating behavior. The interactions are still detectable, but they do not induce a significant change in the NCG electronic structure. The electronic sensitivity of NCG in nucleobase adsorption studies correlates directly with the size of the Dirac point shift. Purine bases induce more significant shifts in the Dirac point, making them more effective in modulating the conductivity of NCG, which is crucial for biosensing applications.

These findings indicate that FET devices can effectively differentiate DNA nucleobases based on their distinct electronic interactions, providing a pathway for highly selective and sensitive biosensing applications.

**Figure 5:**
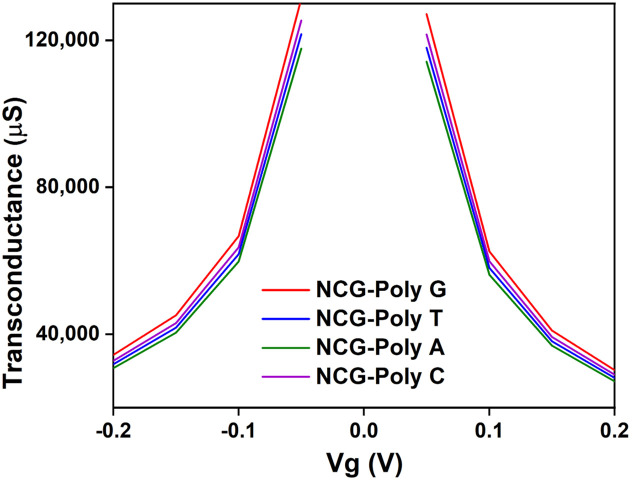
Transconductance of the NCG-FET device after DNA nucleobases immobilization on the NCG channel.

The transconductance behavior (Figure 5) of the NCG-FET devices shows notable changes after DNA nucleobase immobilization, indicating interactions between the nucleobases and the NCG channel. The variations in transconductance suggest that different nucleobases influence charge transport differently, likely due to their distinct electronic properties, indicating specific interactions between each nucleobase and the NCG material. DFT studies show that poly G has the lowest ionization potential (≈7.75 eV), meaning it donates electrons more easily than other nucleobases. This matches the higher transconductance observed experimentally NCG-poly G [51].

Poly G exhibits the strongest interaction with the NCG surface, as it contains a high percentage of oxygen (≈64.89 wt%), contributing to significant π–π stacking interactions with NCG. The high intensity of oxygen-related peaks in the EDAX analysis (641.85) further suggests a robust interaction with the NCG surface. This is also supported by its substantial shift in the electrical characteristics of the device, which indicates strong doping effects and charge transfer to the top graphenic layers. Poly T, while containing a high percentage of oxygen (≈79.54 wt%), has a lower overall carbon content (≈20.44 wt%), resulting in weaker π–π stacking interactions compared with guanine. Its higher error margin (11.12%) for carbon and low nitrogen content (≈0.06 wt%) indicate a less pronounced interaction with the NCG surface, which is reflected in the more moderate changes in the device’s electrical properties. Adenine shows a more balanced composition of carbon (≈37.54 wt%) and oxygen (≈62.62 wt%) but with a lower nitrogen content (≈0.21 wt%), which results in moderate interactions with the NCG channel. The device shows notable changes in electrical behavior, though not as strong as poly G, indicating less effective electron donation to the NCG surface. Cytosine, with a composition similar to guanine (≈33.47 wt% carbon and ≈66.36 wt% oxygen), exhibits moderate interactions with the NCG surface. However, the nitrogen content (≈0.17 wt%) is slightly lower than in guanine, leading to a weaker doping effect. This results in less pronounced modifications in the electrical properties, as seen in the relatively lower net intensity values for carbon and nitrogen (248.06 and 0.49, respectively). These data confirm that guanine induces the most significant modification in the electronic properties of the NCG channel, while thymine and cytosine show weaker interactions, highlighting the important role of nitrogen and oxygen content in modulating the performance of graphene-based biosensors.

**Figure 6:**
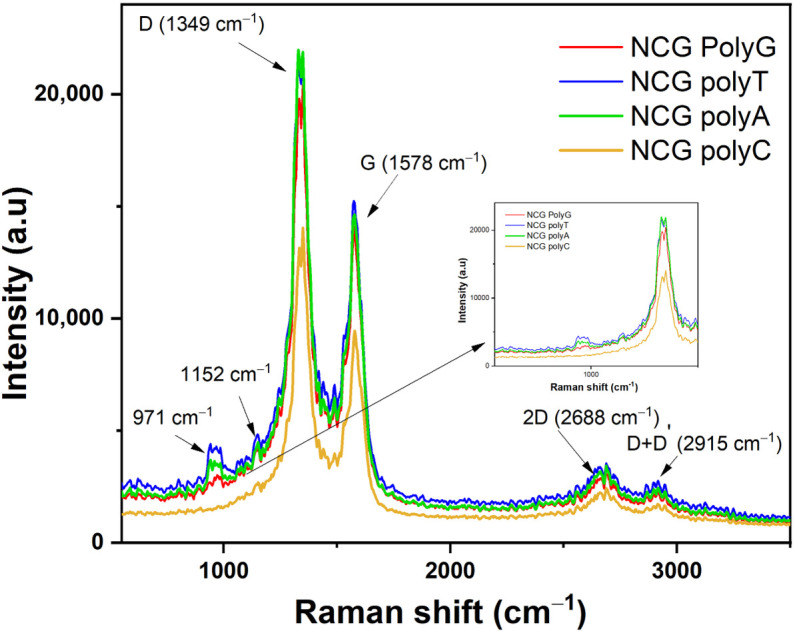
Raman spectra after direct immobilization of DNA nucleobases.

The Raman spectra (Figure 6) also confirm these findings, showing D-band splitting and G-band shifts in all cases, with the purine bases exhibiting more pronounced shifts compared with the pyrimidine bases. These results emphasize the stronger π–π stacking interactions and n-doping effects in adenine and guanine, while thymine and cytosine cause less pronounced structural changes in the graphene lattice.

## 5. Conclusions

Each DNA nucleobase contributes differently to the detection sensitivity of the NCG-FET devices based on its interaction with the NCG channel. The differences in interaction, observed through changes in the Dirac point, transconductance, and Raman spectra, underline the ability of NCG-FETs to differentiate between nucleobases.

Guanine induces the most significant changes in the NCG-FET response, with a pronounced shift in the Dirac point and significant modifications in the Raman spectra and transconductance. Its electron-rich nature, due to the presence of nitrogen atoms, makes it the most effective nucleobase for modulating the electronic properties of the NCG channel. This strong interaction increases the sensitivity of the NCG-FET, allowing for the precise detection of guanine in DNA sequences. Therefore, guanine is the most influential base for improving detection sensitivity in NCG-FET-based biosensors.

Adenine also induces measurable changes in the NCG-FET, though less dramatic than guanine. The Dirac point shift and variations in transconductance are moderate, which suggests a weaker interaction with the NCG channel. However, these changes are still sufficient to contribute to the detection of adenine in a DNA sequence, though with lower sensitivity when compared with guanine. Adenine provides a valuable but less impactful contribution to the detection capabilities of the NCG-FET.

Both thymine and cytosine show relatively weak interactions with the NCG channel, resulting in smaller shifts in the Dirac point and less significant changes in transconductance and Raman spectra. These weaker interactions indicate that thymine and cytosine have a limited impact on the electrical properties of the NCG-FET, leading to lower sensitivity in detecting these bases. However, they still contribute to the overall ability of the NCG-FET to differentiate between nucleobases, though their influence on detection sensitivity is weaker compared with guanine and adenine.

Theoretically, the binding energies of the nucleotide bases are influenced by their electronic structures, with purine bases (guanine and adenine) typically exhibiting stronger binding energies due to their electron-donating properties, which facilitate more robust interactions with graphene or other sensing materials.

In summary, all four nucleobases contribute to the modulation of the NCG channel in FETs. Guanine stands out as having the biggest influence on NCG’s electrical properties. Its strong electronic interaction with the NCG channel makes it a key component for precise and sensitive DNA detection. Adenine, thymine, and cytosine also play roles in detection, but their weaker interactions result in less pronounced effects on the NCG-FET’s electrical properties, contributing to lower sensitivity for detecting these bases.

Current findings pave the way for integrating NCG into FET biosensors. Each nucleobase has its own electric signature and its influence on the electric properties of NCG. By understanding how each of them interacts with the NCG substrate, different types of sensors can be further developed with applications in DNA-based diagnosis, single-nucleotide polymorphism or mutation detection, and even DNA sequencing. Furthermore, artificial intelligence can be trained to analyze the electric signals generated by an NCG-FET sensor so that heterogeneous DNA sequence detection or DNA sequencing is enabled. NCG can also be coupled with CRISPR/Cas molecules to develop new types of biosensors or delivery vectors.

## Figures and Tables

**Table 1:** Analysis of Dirac point shift of the NCG-FETs at different drain potentials, after guanine, thymine, adenine, and cytosine adsorption.

		*V_d_* = 1 V	*V_d_* = 2 V	*V_d_* = 3 V	*V_d_* = −3 V
**Guanine**	V_DP_-NCG1 (V)	1.5	2.7	4.09	−4.05
	V_DP_-NCG1 poly G (V)	1.05	2.09	3.15	−3.15
	**ΔV_DP_ (V)**	**−0.45**	**−0.61**	**−0.94**	**0.9**
**Thymine**	V_DP_-NCG4 (V)	1.15	2.4	3.65	−3.6
	V_DP_-NCG4 poly T (V)	1.05	2.15	3.25	−3.2
	**Δ** **V_DP_ (V)**	**−0.1**	**−0.25**	**−0.4**	**0.4**
**Adenine**	V_DP_-NCG8 (V)	1.2	2.4	3.75	−3.7
	V_DP_-NCG8 poly A (V)	1.1	2.2	3.29	−3.29
	**ΔV_DP_ (V)**	**−0.1**	**−0.2**	**−0.46**	**0.41**
**Cytosine**	V_DP_-NCG12 (V)	1.2	2.45	3.7	−3.7
	V_DP_-NCG12 poly C (V)	1.1	2.2	3.3	−3.25
	**ΔV_DP_ (V)**	**−0.1**	**−0.25**	**−0.4**	**0.45**

**Table 2:** Elemental distribution extracted from EDAX measurements of the NCG FETs’ channel after poly G, poly T, poly A, and poly C adsorption.

	Atoms	Weight %	Atomic %	Net. Int	Error %
**Poly G**	**C**	34.47	41.17	408.10	5.28
	**N**	0.64	0.65	2.92	68.46
	**O**	64.89	58.18	641.85	6.56
**Poly T**	**C**	20.44	25.49	535.7	11.12
	**N**	0.06	0.07	1	99.99
	**O**	79.54	74.4	2326.10	7.17
**Poly A**	**C**	37.54	44.45	319.37	5.21
	**N**	0.21	0.22	0.64	0.0007
	**O**	62.62	55.34	421.42	6.90
**Poly C**	**C**	33.47	40.12	248.06	5.69
	**N**	0.17	0.17	0.49	90.67
	**O**	66.36	59.71	423.55	6.74

## Data Availability

The data presented in this study are available on request from the corresponding author.
